# Hepatic Resection Using a Water Jet Dissector

**DOI:** 10.1155/1993/82362

**Published:** 1993

**Authors:** H. U. Baer, S. C. Stain, T. Guastella, G. J. Maddern, L. H. Blumgart

**Affiliations:** Clinic for Visceral and Transplantation Surgery Inselspital University of Berne Berne CH-3010 Switzerland

## Abstract

The mortality and morbidity in major hepatic resection is often related to hemorrhage. A high pressure, high velocity water jet has been developed and has been utilized to assist in hepatic parenchymal transection. Sixty-seven major hepatic resections were performed for solid hepatic tumors. The tissue fracture technique was used in 51 patients (76%), and the water jet dissector was used predominantly in 16 patients (24%). The extent of hepatic resection using each technique was similar. The results showed no difference in operative duration (*p* = .499). The mean estimated blood loss using the water jet was 1386 ml, and tissue fracture technique 2450 ml (*p* = .217). Transfusion requirements were less in the water jet group (mean 2.0 units) compared to the tissue fracture group (mean 5.2 units); (*p* = .023). Results obtained with the new water dissector are encouraging. The preliminary results suggest that blood loss may be diminished.


HPB Surgery, 1993, Vol. 6, pp. 189–198
Reprints available directly from the publisher
Photocopying permitted by license only

© 1993 Harwood Academic Publishers GmbH
Printed in the United States of America

### HEPATIC RESECTION USING A WATER JET DISSECTOR

H.U. BAER, S.C. STAIN, T. GUASTELLA, G.J. MADDERN and L.H. BLUMGART

*Clinic for Visceral and Transplantation Surgery, Inselspital, University of Berne, CH-3010 Berne, Switzerland*

*(Received 22 April 1992)*

The mortality and morbidity in major hepatic resection is often related to hemorrhage. A high pressure, high velocity water jet has been developed and has been utilized to assist in hepatic parenchymal transection. Sixty-seven major hepatic resections were performed for solid hepatic tumors. The tissue fracture technique was used in 51 patients (76%), and the water jet dissector was used predominantly in 16 patients (24%). The extent of hepatic resection using each technique was similar. The results showed no difference in operative duration ($p = .499$). The mean estimated blood loss using the water jet was 1386 ml, and tissue fracture technique 2450 ml ($p = .217$). Transfusion requirements were less in the water jet group (mean 2.0 units) compared to the tissue fracture group (mean 5.2 units); ($p = .023$).

Results obtained with the new water dissector are encouraging. The preliminary results suggest that blood loss may be diminished.

KEY WORDS: Hepatic resection, water jet dissector, liver tumour

### INTRODUCTION

Major hepatic resection can be performed with low operative mortality and postoperative morbidity. Mortality is often related to operative hemorrhage, and the most common complications after operation are postoperative bleeding, biliary fistula, or intraabdominal abscess. Various methods have been developed to assist the hepatic parenchymal dissection by the exposure of the intact vascular elements and bile ducts for secure ligation. Tissue fracture using either the fingers and/or a Kelly clamp are the most common methods[1]. A new approach for parenchymal dissection was reported by Papachristou in 1982 using a jet of water to disrupt the liver tissue and display the blood vessels and bile ducts[2]. Since that time, other investigators have introduced various models of water jet apparatus[3,4].

We have been concerned in the development of a high pressure, high velocity water jet which offers several advantages over previous models (ME Medical Exports AG, Zug, Switzerland)[5,6]. This device has been in clinical use for two years, and has been employed for hepatic resection and the intrahepatic exposure of bile ducts for biliary enteric anastomoses. This report examines the results obtained using the traditional tissue fracture technique compared to those obtained

Address correspondence to: Hans U. Baer, M.D., Clinic for Visceral and Transplantation Surgery, Inselspital, University of Berne, CH-3010 Berne, Switzerland recently with the addition of the water jet dissector in major resection for solid hepatic tumors. During this time period, we have instituted other improvements in technique. These have included: the control of central venous pressure during hepatic transection so as to limit hepatic venous hemorrhage, and the use of fewer perihepatic drains in most cases for only 24 hours.

### MATERIALS AND METHODS

Sixty-seven major hepatic resections were performed for solid tumors over a four year period (October 1986 to October 1990) at the Clinic for Visceral and Transplantation Surgery, Inselspital, University of Bern. There were 35 males, and 32 females, with a mean age of 56 years (26 -- 48 years). The diagnoses of these patients were: metastatic tumors [38], primary hepatocellular carcinoma [15], hilar cholangiocarcinoma [11], fibroadenoma [2], and focal nodular hyperplasia [1]. Resections performed for Hydatid disease, hemangioma, and polycystic liver disease were excluded. The tissue fracture technique was used exclusively in 51 (76%). The water jet was used predominantly in 16 hepatic resections (24%). Patients with atypical resections included those in whom at least one hepatic segment was removed (mean 1.8 segments; range 1 -- 3).

The groups were not randomized. However the water jet was used almost exclusively during the last 18 months of the study period except when not available due to minor design modifications. Clinic records were reviewed to determine duration of operation, estimated blood loss (EBL), transfusion requirements, postoperative biliary fistulas, and intraabdominal abscesses. Operative blood loss (EBL) was estimated by the anesthetist. The water jet uses 200 – 300 ml of saline in a major parenchymal transection. This fluid was aspirated with the blood loss into the suction cannister, and was not deducted from the EBL. Transfusion totals were compiled from the requirements during the entire hospitalization.

The entire groups of patients operated on using the water jet and tissue fracture methods were compared using the Kruskall - Wallis one way analysis of variance. The small sample size did not allow statistical comparison for the individual operative procedures. The two methods were however, analyzed specifically for comparison of the more extensive resections. Right hepatectomy, extended right hepatectomy, or extended left hepatectomy are generally more lengthy operative procedures, associated with increased operative blood loss and for purposes of analysis were regarded as extensive. Twelve of the 16 patients in the water jet group (75%) had these more extensive procedures, compared to 30 of the 51 patients in the tissue fracture group (59%). These proportions were statistically similar (Chi square = 1.071; $p$ = .301).

### RESULTS

The duration of operation using the water jet dissector was a mean of 285 minutes (range 160 -- 480), and with the tissue fracture technique a mean of 272 minutes (range 120 -- 540). This difference was not significant ($p$ = .499). Also in the more extensive procedures, there was not a significant difference in operative duration ($p$ = .728). The times of the individual procedures are listed in Table 1.

**Table 1**

| Operation | Method (N) | Operative time Mean | Median | Range |
|---|---|---|---|---|
| Right hepatectomy | water jet (3) | 277 min | 270 min | (240-320 min) |
| | tissue (11) | 299 min | 300 min | (225-390 min) |
| Ext R hepatectomy | water jet (7) | 292 min | 270 min | (225-380 min) |
| | tissue (15) | 345 min | 240 min | (180-460 min) |
| Ext L hepactectomy | water jet (2) | 398 min | 398 min | (315-480 min) |
| | tissue (4) | 360 min | 330 min | (250-540 min) |
| Left hepatectomy | water jet (2) | 275 min | 275 min | (260-290 min) |
| | tissue (5) | 256 min | 240 min | (180-360 min) |
| Atypical resection | water jet (2) | 170 min | 170 min | (160-180 min) |
| | tissue (16) | 226 min | 195 min | (120-480 min) |

The hemoglobin levels at admission were comparable. In the water jet group the mean hemoglobin was 12.9 g/dl (range 9.3 -- 16.0), and in the tissue fracture group the mean was 12.8 g/dl (range 9.2 -- 16.7). The EBL and number of units of blood transfused are listed in Tables 2 and 3. In the water jet group the mean EBL was 1386 ml (median 1200, range 300 -- 3000 ml) and using the tissue fracture technique the mean was 2450 ml (median 1600, range 100 – 12,000 ml). Although there was a trend towards decreased blood loss using the water jet, the difference was not significant either in the entire group ($p$ = .217), or in the more extensive resections ($p$ = .106).

Transfusion requirements are depicted in Figure 1. Patients in whom the water jet was used had a mean blood transfusion of 2.0 units (median 1.5, range 0 – 8 units); and with the tissue fracture method, the mean transfusion was 5.2 units (median 3.5, range 0 -- 34 units). This difference was significantly less using the water jet ($p$ = .023), and this also held true for the more extensive procedures ($p$ = .025).

A biliary fistula was defined as an abnormal drainage of fluid with a bilirubin level greater than that in the serum, and which persisted for more than 7 days[7]. This occurred in one case after a right hepatectomy performed with the water jet. Three patients had postoperative intrabdominal fluid collections with positive bacterial cultures. All three occurred using the water jet and were treated by CT guided percutaneous catheter drainage. Clinically insignificant fluid collections which did not require specific therapy, or were sterile on aspiration were identified in eight patients (water jet -1; tissue fracture -7).

Five patients died in hospital or within 30 days (7.5%). The tissue fracture technique was used in four and the water jet in one. The causes of death were pulmonary embolism, postoperative hemorrhage due to coagulopathy, multisystem organ failure, hepatic necrosis after hepatic arterial ligation, and liver failure in a patient who previously had received multiple courses of hepatic arterial infusion of chemotherapy. In this patient, the intrahepatic vessels were thrombosed.

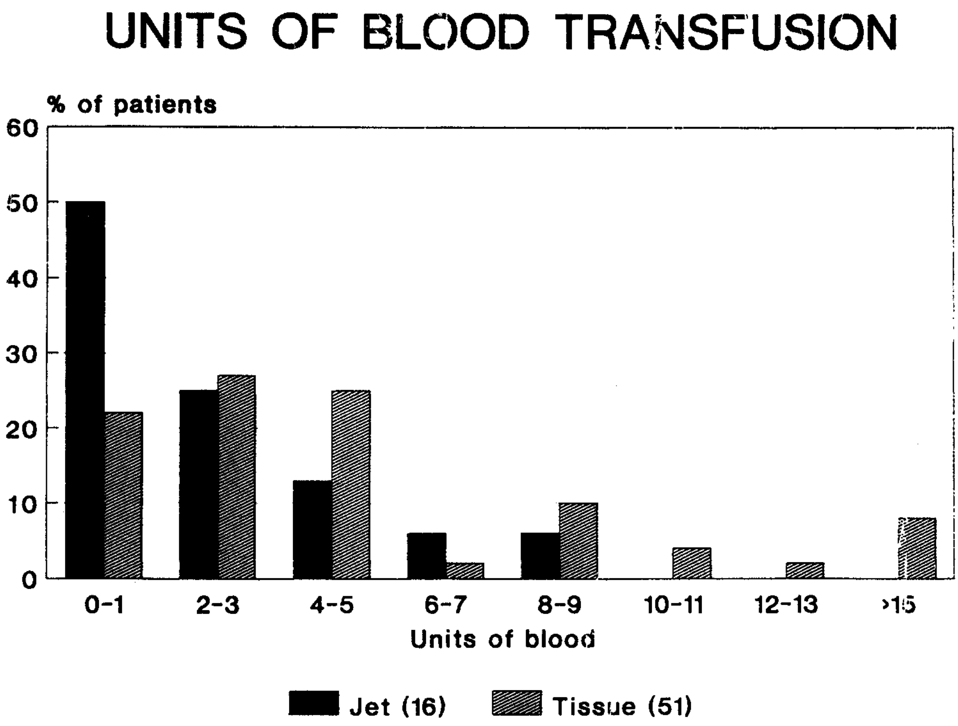

**Figure 1** Requirements of blood transfusion during hospitalization.

**Table 2**

| Operation | Method (N) | Estimated Blood Loss[a] Mean | Median | Range |
|---|---|---|---|---|
| Right hepatectomy | water jet (3) | 1400 ml | 1200 ml | (1000-1200 ml) |
| | tissue (11) | 2311 ml | 2000 ml | (800-6000 ml) |
| Ext R hepatectomy | water jet (7) | 1657 ml | 1500 ml | (600-3000 ml) |
| | tissue (15) | 3347 ml | 1800 ml | (100-12000 min) |
| Ext L hepactectomy | water jet (2) | 1000 ml | 1000 ml | (1000 ml) |
| | tissue (4) | 2667 ml | 3000 ml | (1500-3000 ml) |
| Left hepatectomy | water jet (2) | 1100 ml | 1100 ml | (1000-1200 ml) |
| | tissue (5) | 1720 ml | 900 ml | (200-3500 ml) |
| Atypical resection | water jet (2) | 900 ml | 900 ml | (300-1500 ml) |
| | tissue (16) | 1667 ml | 1500 ml | (300-5600 min) |

[a]Approximately 300 cc of aspirated jet fluid not deducted from EBL in water jet group.

**Table 3**

| Operation | Method (N) | Units of Blood Transfused | | |
|---|---|---|---|---|
| | | Mean | Median | Range |
| Right hepatectomy | water jet (3) | 0.7 | 1 | (0-2) |
| | tissue (11) | 4.3 | 4 | (0-10) |
| Ext R hepatectomy | water jet (7) | 2.7 | 2 | (0-8) |
| | tissue (15) | 8.5 | 4 | (0-34) |
| Ext L hepactectomy | water jet (2) | 4.0 | 4 | (4) |
| | tissue (4) | 8.8 | 8.5 | (2-16) |
| Left hepatectomy | water jet (2) | 1.5 | 1.5 | (0-3) |
| | tissue (5) | 1.2 | 0 | (0-4) |
| Atypical resection | water jet (2) | 0 | 0 | (0) |
| | tissue (16) | 3.1 | 2 | (0-11) |

### DISCUSSION

Hepatic resection has become a commonly performed procedure for both primary and metastatic tumors. In several large series, operative mortality and morbidity has been related to blood loss and operative duration[8,9]. The reported mortality in specialized hepatobiliary centers approaches 5%[10,11,12], and efforts to improve the safety of resection have largely centered upon the control of hemorrhage during parenchymal transection. All of these methods have been directed at the intact isolation of the vascular elements for ligation; and in particular, the control of hepatic venous hemorrhage.

The finger fracture technique was introduced by Anschutz in 1903[13]. It was popularized by Lin, who utilized a clamp to crush the parenchyma, exposing the vessels and bile ducts for ligation[1]. There have since been many efforts to improve upon this method including the suction knife, YAG laser, and the ultrasonic dissector[14,15,16,17]. The latter device has been widely adopted, although few studies have compared its efficiency versus traditional tissue fracture. Little recently reported an improvement in his own results of hepatic resection using a combination of ultrasonic dissector and intraoperative ultrasound, although the majority of the resections were of only one or two hepatic segments[18]. Our own experience using the ultrasonic dissector has been with an older model, the CUSA 100 (Cavitron, Inc., Stanford, Conn.), but some surgeons have found CUSA to be slow, cumbersome and ineffective in fibrous tissue[15,18,19]. The tissue fracture technique remains the least expensive and most common method employed for hepatic resection.

Papachristou utilized a commercially available agricultural electric sprayer which disrupted the parenchyma, and exposed the vessels[2]. Several authors have since reported experimental and clinical use of specially designed water jets[3,4,20]. In one study, Une *et al.* compared the ultrasonic dissector to their model of a water jet, and although there was not a difference in blood loss, the operative time was prolonged in the ultrasonic dissector group[20].

During recent years, we have improved our method of hepatic transection, and in addition to the water jet, we have maintained the central venous pressure at approximately 5 cm water pressure during the parenchymal dissection phase, so as to decrease hepatic venous pressure and reduce hepatic venous blood loss. The high pressure, high velocity water jet dissector employed in this study has been described previously[6] including preliminary results[5]. It has fundamental differences from other models. The instrument consists of a hydrodynamic device attached to compressed air which delivers pressurized liquid to a fine tipped nozzle (Figure 2). The specially designed nozzle has a smaller diameter than those used previously, and delivers less fluid at a higher velocity and pressure than the models reported previously[3,4]. While other authors noted that 1500 ml of fluid was used by their device in a standard right lobectomy our water jet typically uses 200 -- 300 ml for a major hepatic resection. The ultra thin nozzle tip in our water jet produces a stream which is coherent for only 30 mm, and then radiates into a divergent spray of microdroplets. The jet can effectively cut hepatic parenchyma, including fibrous tissue, and the spray washes away the debris to display the vessels and bile ducts for ligation. The divergent spray also allows the surgeon to vary the cutting potential by simply altering the distance between the nozzle tip and the parenchyma.

Operative time using the water jet dissector and tissue fracture technique were similar. Blood loss estimates are inherently inaccurate, although there was a trend toward lower estimated blood loss, more pronounced in the more extensive resections. The significant decrease in transfusion requirement may be a more

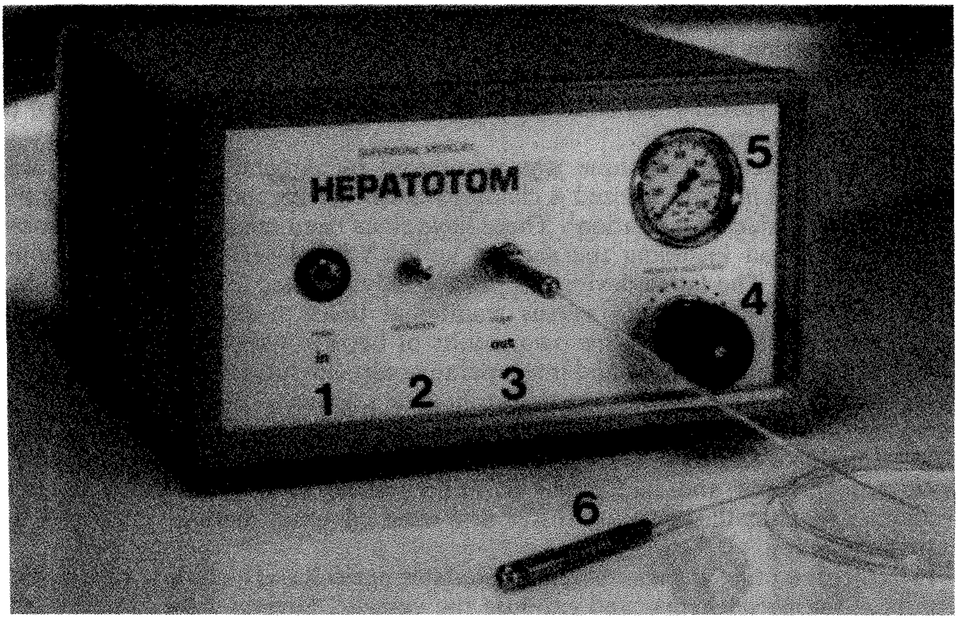

**Figure 2** Water jet dissector (ME Medical Exports AG, Zug, Switzerland) with: (1) Fluid inlet port (2) Activation connection (3) Probe connection (4) Pressure adjustment knob (5) Pressure indicator (6) Connected probe.

important parameter. A decrease in operative hemorrhage however is only one measure of efficacy. Subjectively, we believe the water jet is especially useful in the removal of very large tumors which encroach upon the main hepatic veins. Resection was made possible in some cases where only a minimal clearance was feasible. The method is useful in extended left hepatectomy where intrahepatic exposure of the right anterior sectoral bile duct and preservation of the remaining posterior sectoral duct is essential.

The increased incidence of intraabdominal abscesses in the water jet group may be of concern. However, two of the three patients who developed abscesses had obstructive jaundice due to hilar cholangiocarcinoma, and the bile already had a positive culture at the time of operation. Hepatic resection in the presence of obstructive jaundice has been shown to have an increased incidence of infectious complications[21,22]. The sterility of the system has previously been tested by overnight incubation with Proteus, followed by bacterial culture after standard sterilization procedures[5]. No organisms were recovered. The addition of bacteriostatic antibiotics to the jet fluid is possible, and is currently under investigation.

Splashing of water has been reported to be a problem in previous models of water jets[2,4]. With the fine microdroplets in this system we have not observed splashing. A recent report using the ultrasonic dissector for hepatic resection in eight patients with positive Hepatitis B surface antigen (HBsAg) showed that in seven patients, the fluid aspirated by the ultrasonic dissector was also HBsAg positive[23]. This is not surprising because the shed blood is also aspirated. There are potential risks to the operating room personnel whenever patients are operated upon with hepatitis or other viral diseases. We agree with the recommendations that eye shields and protective gowns and masks should be used when there is the potential for splashing of body fluids and realistically that includes any operative procedure[23,24].

In summary, we have found the high pressure, high velocity water jet dissector a valuable adjunct, especially in large or extended hepatic resections. The device is simple in concept, rapid to use, and the technique is easily learned. Transfusion requirements were significantly decreased, and with a larger sample size and more precise documentation of blood loss the estimated blood loss may also be significantly diminished. The water jet is not, however, a replacement for a thorough knowledge of hepatic segmental anatomy and the relationship of the branches of the hepatic artery, veins, portal veins and bile ducts. Our experience with the high pressure, high velocity water jet in hepatic resection is encouraging. The device has also proved useful in the facilitation of intrahepatic dissection to expose peripheral or segmental bile ducts for palliative biliary enteric bypass. Modifications of the system are under development for neurosurgical, urological, and laparoscopic applications.

### *References*

1. Lin, T.Y. (1983) Results in 107 hepatic lobectomies with preliminary report on the use of a clamp to reduce blood loss. *Ann. Surg.*, **177**, 413–421
2. Papachristou, D.N. and Barters, R. (1982) Resection of the liver with a water jet. *Br.J.Surg.*, **69**, 93–94
3. Uchino, J., Une, Y., Horie, T., Yonekawa, M., Kakita, A. and Sano, F. (1988) Surgical cutting of the liver by water jet. Sendai, Ninth International Symposium on Jet Cutting Technology. BHRA, The Fluid Engineering Centre, Cranfield, Bedford, UK. pp. 629–639

4. Persson, B.G., Jeppsson, B., Tranberg, K.G., Roslund, K. and Bengmark, S. (1988) Transection of the liver with a water jet. *Surg.Gynecol.Obstet.*, **168**, 267–268
5. Baer, H.U., Maddern, G.J. and Blumgart, L.H. (1991) Hepatic surgery facilitated by a new jet dissector. *HPB Surg.*, **4**, 137–146
6. Baer, H.U., Maddern, G.J. and Blumgart, L.H. (1991) New water-jet dissector: initial experience in hepatic surgery. *Br.J.Surg.*, **78**, 502–503
7. Czerniak, A., Thompson, J.N., Soreide, O., Benjamin, I.S. and Blumgart, L.H. (1988) The management of fistulas of the biliary tract after injury to the bile duct during cholecystectomy. *Surg.Gynecol.Obstet.*, **167**, 33–38
8. Andersson, R., Saarela, A., Tranberg, K.G. and Bengmark, S. (1990) Intraabdominal abscess after major liver resection. *Acta Chir.Scand.*, **156**, 707–710
9. Adson, M.A. (1988) Primary hepatocellular cancer - Western experience. In Surgery of the liver and biliary tract, edited by L.H. Blumgart, vol. II, pp. 1153–1165. Edinburgh, London, Melbourne, New York: Churchill Livingstone
10. Adson, M.A. and Weiland, L.H. (1981) Resection of primary solid hepatic tumors. *Am.J.Surg.*, **141**, 18–21
11. Iwatsuki, S. and Starzl, T.E. (1988) Personal experience with 411 hepatic resections. *Ann.Surg.*, **208**, 421–434
12. Schwartz, S.I. (1990) Hepatic resection. *Ann.Surg.*, **211**, 1–8
13. Anschutz, W. (1903) Ueber die Resektion der Leber. *Sant.K.Vort.* 356–357
14. Haapiainen, R. and Schröder, T. (1989) The suction knife in liver surgery. *Am.J.Surg.*, **157**, 340–342
15. Mason, G.R. (1989) Hepatic resection technique. *Am.J.Surg.*, **157**, 343–345
16. Putnam, C.W. (1989) Techniques of ultrasonic dissection in resection of the liver. *Surg.Gynecol.Obstet.*, **157**, 475–478
17. Hodgson, W.J.B. and Del Guercio, L.R.M. (1984) Preliminary experience in liver surgery using the ultrasonic scalpel. *Surgery*, **95**, 230–234
18. Adson, M.A. In discussion: Fiddian-Green, R.G., Siviski, P.R., Karol, S.V. (1988) Median hepatectomy using ultrasonic dissection for complex hepatobiliary problems. *Arch.Surg.*, **123**, 901–907
19. Little, J.M. and Hollands, M.J. (1991) Impact of the CUSA and operative ultrasound on hepatic resection. *Hepatobiliary Surg.*, **3**, 271–778
20. Une, Y., Uchino, J., Horie, T., Sato, Y., Ogawawara, K., Kakita, A. and Sano, F. (1989) Liver resection using a water jet. *Cancer Chemother.Pharmacol.*, 23 Suppl, S74–S77
21. Edwards, W.H. and Blumgart, L.H. (1987) Liver resection in malignant disease. *Semin.Surg.Oncol.*, **3**, 1–11
22. Pace, R.F., Blenkharn, J.I., Edwards, W.J., Orloff, M., Blumgart, L.H. and Benjamin, I.S. (1989) Intra-abdominal sepsis after hepatic resection. *Ann.Surg.*, **209**, 302–306
23. Matsumata, T., Kanematsu, T., Okadome, K. and Sugimachi, K. (1991) Possible transmission of serum hepatitis in liver surgery with the ultrasonic dissector. *Surgery*, **109**, 284–285
24. Hammond, J.S., Eckes, J.M., Gomez, G.A. and Cunningham, D.N. (1990) HIV, trauma and infection control: Universal precautions are universally ignored. *J.Trauma*, **30**, 555–561

*(Accepted by S. Bengmark 19 May 1992)*

### INVITED COMMENTARY

The authors have been involved in the development of a water jet dissector and reported their initial experience in 1991. They now present the outcome of 19 liver transections using the water jet dissector and compare the results with a group of 51 liver resections in which transections were made by tissue fracture. Although the groups were not randomized they seem to be fairly matched. The duration of trnsection was the same but transfusion requirements were reduced when the water jet was used. Of major concern was the occurrence of biliary fistula (1 patient) and abscesses (3 patients) in the water jet group.

By reducing hemorrhage in a major liver transection recovery is improved and postoperative complications are less likely to follow. This is even more important as recurrence, to some extent, may be associated with increased transfusions. The water jet is one of several tools that may aid in a liver resection. The design of the beam may well be of importance; a more coherent jet compared to a more spray-like jet. By adding vasoactive substances haemostasis may be improved. Although a well controlled resection can be performed hazardous bleeding may be diminished when a water jet or an ultrasonic dissector is used. Preference for either of these tools is more a matter of temperament; the water jet being more speedy. The cost of these dissectors is of course of importance; a water jet uses a simpler technique and therefore appears to be less costly.

Difficulties in sterilization is the main disadvantage of current water jet dissectors. The water passes through a pumpsystem that is not autoclavable or single-use and has to be sterilized after each use by flooding ethanol or other detergents through it. This procedure may well be sufficient in the hands of the inventors but inappropriate when it is more generally applied at other institutions. The reported increased incidence of abscesses, in the present study, may well be caused by bacterial growth in the pumpsystem. The addition of antibacterial drugs as proposed by the authors is dangerous because a selective growth of more virulent bacteria may well take place. Therefore, sterilization must be controlled before marketing water jet machines.

B.O.Persson
Lund University
Lund, Sweden

### INVITED COMMENTARY

Baer and his colleague have been successfully working on the development of a new water jet which seems to have overcome many of the problems previously encountered with this method of dividing hepatic parenchyma. By using a coherent stream of water which is powerful only up to 3 cm, the hepatic parenchyma can be effectively cut. Only a small volume of water is necessary and this reduces the major contraindication of water jets in the past where large volumes of water were required. Also, the microdroplets produced by Baer's instrument appear to reduce splashing.

Experience is presented on sixteen cases but it is of concern that three of them developed intra-abdominal abscesses.

The time for parenchymal dissection using this technique was about the same as finger fracture but blood loss was halved to a mean of 1,386 ml which is still more than lost by using the ultrasonic aspirator. Indeed, the CUSA system, because of its worldwide use, has become the standard against which other techniques are measured. Addition of electrocautery to the CUSA system may make this technique more efficient and faster.

The water jet is rapidly improving, and Baer and his colleagues will no doubt continue to refine it.

### *Reference*

Hodgson, W.J.B., Morgan, J., Byrne, D. and DelGuercio, L.R.N. (1992) Hepatic Resections for Primary and Metastatic Tumors Using the Ultrasonic Surgical Dissector. *Amer.Jour.of Surgery*, **163**, 246–250

W. John B. Hodgson, M.D.
Chief of GI Surgery
Professor of Surgery
New York Medical College, USA

